# Supplementary material for: Regeneration of a full-thickness defect in rotator cuff tendon with umbilical cord-derived mesenchymal stem cells in a rat model
Source: PLoS One. 2020 Nov 9;15(11):e0235239. doi: 10.1371/journal.pone.0235239 (PMC7652329; doi:10.1371/journal.pone.0235239)
Supplement: S2 Table — (DOCX) [file pone.0235239.s002.docx]

**S2 Table. Macroscopic evaluation of tendon regeneration**

| **Parameters** | **2 weeks** | | | | **4 weeks** | | | |
| --- | --- | --- | --- | --- | --- | --- | --- | --- |
|  | **Control** | **Saline** | **UC MSCs** | **p value** | **Control** | **Saline** | **UC MSCs** | **p value** |
| Tendon rupture | 0.00 ± 0.00 | 0.00 ± 0.00 | 0.00 ± 0.00 | n.a. | 0.00 ± 0.00 | 0.00 ± 0.00 | 0.00 ± 0.00 | n.a. |
| Inflammation | 0.00 ± 0.00 | 1.00 ± 0.00 | 0.75 ± 0.50 | a, b | 0.00 ± 0.00 | 1.00 ± 0.00 | 0.25 ± 0.50 | a, c |
| Tendon surface | 0.00 ± 0.00 | 1.00 ± 0.00 | 1.00 ± 0.00 | n.a. | 0.00 ± 0.00 | 0.00 ± 0.00 | 0.00 ± 0.00 | n.a. |
| Neighbouring tendon | 0.00 ± 0.00 | 0.50 ± 0.58 | 0.00 ± 0.00 | n.s. | 0.00 ± 0.00 | 0.50 ± 0.58 | 0.00 ± 0.00 | n.s. |
| Level of the defect | 0.00 ± 0.00 | 0.25 ± 0.50 | 0.00 ± 0.00 | n.s. | 0.00 ± 0.00 | 0.00 ± 0.00 | 0.00 ± 0.00 | n.a. |
| Defect size | 0.00 ± 0.00 | 0.50 ± 0.58 | 0.00 ± 0.00 | n.s. | 0.00 ± 0.00 | 0.75 ± 0.50 | 0.00 ± 0.00 | a, c |
| Swelling/redness of tendon | 0.00 ± 0.00 | 1.25 ± 0.50 | 1.25 ± 0.50 | a, b | 0.00 ± 0.00 | 1.00 ± 0.00 | 0.25 ± 0.50 | a, c |
| Connection surrounding tissue and slidability | 0.00 ± 0.00 | 1.00 ± 0.00 | 0.75 ± 0.50 | a, b | 0.00 ± 0.00 | 1.00 ± 0.00 | 0.00 ± 0.00 | n.a. |
| Tendon thickness (shape of tendon) | 0.00 ± 0.00 | 2.50 ± 0.58 | 1.50 ± 0.58 | a, b, c | 0.00 ± 0.00 | 2.00 ± 0.00 | 1.00 ± 0.00 | n.a. |
| Color of tendon | 0.00 ± 0.00 | 1.00 ± 0.00 | 1.00 ± 0.00 | n.a. | 0.00 ± 0.00 | 1.00 ± 0.00 | 1.00 ± 0.00 | n.a. |
| Single strains of muscle | 0.00 ± 0.00 | 0.00 ± 0.00 | 0.00 ± 0.00 | n.a. | 0.00 ± 0.00 | 0.00 ± 0.00 | 0.00 ± 0.00 | n.a. |
| Transition of the construct to the surrounding healthy tissue | 0.00 ± 0.00 | 1.00 ± 0.00 | 1.00 ± 0.00 | n.a. | 0.00 ± 0.00 | 1.00 ± 0.00 | 1.00 ± 0.00 | n.a. |
| Total | 0.00 ± 0.00 | 10.00 ± 1.15 | 7.25 ± 0.96 | a, b, c | 0.00 ± 0.00 | 8.25 ± 0.96 | 3.50 ± 1.00 | a, b, c |

Data are presented as mean ± standard deviation. Statistically significant differences (p < .05) are denoted as follows; a: control vs saline, b: control vs UC MSCs, c: saline vs UC MSCs. Abbreviations: UC MSCs, umbilical cord derived mesenchymal; n.a., not available; n.s.; not significant.
